# Lactate concentration in breast cancer using advanced magnetic resonance spectroscopy

**DOI:** 10.1038/s41416-020-0886-7

**Published:** 2020-05-19

**Authors:** Sai Man Cheung, Ehab Husain, Yazan Masannat, Iain D. Miller, Klaus Wahle, Steven D. Heys, Jiabao He

**Affiliations:** 1 Institute of Medical Sciences, School of Medicine, University of Aberdeen, Aberdeen, UK; 2 Pathology Department, Aberdeen Royal Infirmary, Aberdeen, UK; 3 Breast Unit, Aberdeen Royal Infirmary, Aberdeen, UK; 4 Strathclyde Institute of Pharmacy and Biological Sciences, University of Strathclyde, Glasgow, UK

**Keywords:** Prognostic markers, Breast cancer

## Abstract

**Background:**

Precision medicine in breast cancer demands markers sensitive to early treatment response. Aerobic glycolysis (AG) upregulates lactate dehydrogenase A (LDH-A) with elevated lactate production; however, existing approaches for lactate quantification are either invasive or impractical clinically.

**Methods:**

Thirty female patients (age 39–78 years, 15 grade II and 15 grade III) with invasive ductal carcinoma were enrolled. Lactate concentration was quantified from freshly excised whole tumours with double quantum filtered (DQF) magnetic resonance spectroscopy (MRS), and Nottingham Prognostic Index (NPI), LDH-A and proliferative marker Ki-67 were assessed histologically.

**Results:**

There was a significantly higher lactate concentration (*t* = 2.2224, *p* = 0.0349) in grade III (7.7 ± 2.9 mM) than in grade II (5.5 ± 2.4 mM). Lactate concentration was correlated with NPI (*ρ* = 0.3618, *p* = 0.0495), but not with Ki-67 (*ρ* = 0.3041, *p* = 0.1023) or tumour size (*r* = 0.1716, *p* = 0.3645). Lactate concentration was negatively correlated with LDH-A (*ρ* = −0.3734, *p* = 0.0421).

**Conclusion:**

Our results showed that lactate concentration in whole breast tumour from DQF MRS is sensitive to tumour grades and patient prognosis.

## Background

The 10-year survival rate of breast cancer has improved substantially from 40.1 to 78.4% in the last 40 years,^1^ primarily resulting from advancement in chemotherapy^2^ and targeted endocrine modulation.^3^ Non-responding patients not only are exposed to side effects and possible life threatening complications,^4^ but also may experience disease progression during treatment and delayed surgery,^5^ demanding an early response marker beyond crude tumour size. Aerobic glycolysis (AG), a central feature of tumour metabolism, fuels rapid growth with elevated glucose consumption and subsequent lactate production.^6^ Malignant transformation is underscored by genetic mutation, activating hypoxia inducible factor 1 (HIF-1) and subsequent upregulation of lactate dehydrogenase A (LDH-A), leading to increased conversion of pyruvate to lactate and the efflux of lactate through monocarboxylate transporter 4 (MCT4).^7^ The accumulation of lactate generates an acidic environment suppressing normal immunological functions while providing fuel to tumour cells, resulting in accelerated local invasion.^8^ The inhibition of lactate production induces tumour regression in cancer xenografts, underpinning the role of lactate concentration as a response marker.^9^

Biochemical approaches, although capable of lactate concentration quantification, are performed on excised tissue or biopsy samples with limited spatial extent. ^18^F-Fluorodeoxyglucose (FDG) positron emission tomography (PET) sensitive to glucose uptake, suffers from the dependency on glucose delivery and exposure of patients to ionising radiation.^10^ Hyperpolarised ^13^C-pyruvate magnetic resonance spectroscopic imaging maps the conversion of pyruvate to lactate, but suffers from the high cost of labelled pyruvate and specialist equipment.^11^ Conventional ^1^H magnetic resonance spectroscopy (MRS) allows lactate quantification in neuro-oncology,^12^ but suffers from overwhelming contamination signal from lipid in breast. Double quantum filtered (DQF) MRS, a method recently available for clinical research,^13^ allows lactate quantification in high lipid tissue such as breast using standard hardware, but was so far only examined in proof of concept case studies.^14^

We therefore hypothesise that lactate concentration derived from DQF MRS is sensitive to tumour grades and is associated with prognosis.

## Methods

**Fig. 1:**
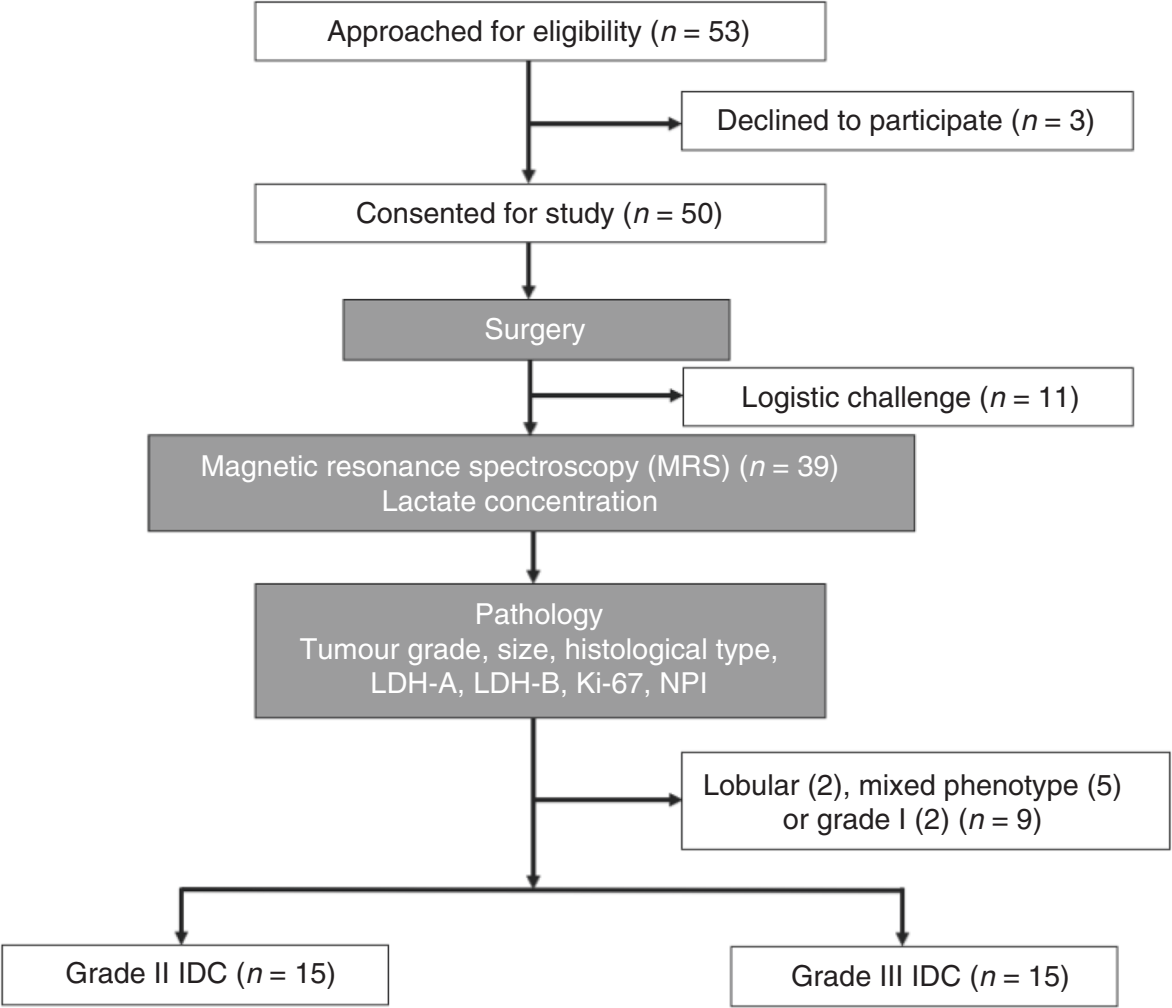
Study design. The study adopted a two-group cross sectional arrangement as shown in this flow chart. Among 53 consecutive patients identified and approached, 50 patients were consented and received wide local excision or mastectomy. After surgery, the freshly excised tumours were scanned on a clinical 3 T MRI scanner to derive lactate concentration within the whole tumour using double quantum filtered (DQF) magnetic resonance spectroscopy (MRS). Subsequently, histopathological analysis provided tumour grade, size, histological type, lactate dehydrogenase A and B (LDH-A and LDH-B), Ki-67 expression and Nottingham Prognostic Index (NPI). Thirty patients with invasive ductal carcinoma (IDC) (15 grade II and 15 grade III) were eventually entered into the study and used for all statistical analysis.

To probe the hypothesis, we conducted a two-group cross sectional study examining the lactate concentration of whole tumour freshly excised from patients, with further comparison against histopathological findings (Fig. 1).

### Study approval

The study was approved by the North West–Greater Manchester East Research Ethics Committee (REC Reference: 16/NW/0032), and signed written informed consent was obtained from the patients prior to inclusion in the study.

### Patients

Thirty female patients (age 39–78 years, 15 grade II and 15 grade III) with invasive ductal carcinoma were enrolled in the study. Only patients undergoing wide local excision or mastectomy, and with a tumour size larger than 1 cm in diameter on ultrasound were eligible. Patients with previous breast malignancies or who had undergone prior neoadjuvant chemotherapy or neoadjuvant hormonal therapy were not eligible. In total, 50 patients consented to be included in the study from 53 consecutive patients in the Breast Unit at Aberdeen Royal Infirmary. From these 50 resected tumour specimens, 39 were scanned, with the remainder excluded because of either theatre delays or MRI scanner unavailability. Following histopathological examination of the excised tumour, alterations in tumour subtype or grade from biopsy that classified the tumour differently from grade II/III invasive ductal in type were excluded. In total, nine tumour specimens, including two lobular phenotype, five mixed phenotype and two grade I, were excluded.

Power calculation was based on Cohen’s *d*,^15^ using the reported difference in lactate concentration of 2 mM between aggressive and non-aggressive tumours in xenografted mice models,^16^ and standard deviation (SD) of 3 mM determined from repeated phantom experiment. Therefore, to show a significant mean difference between groups at the 5% level with 80% power using an independent sample design would require 15 subjects per group.^17^

### Magnetic resonance

After wide local excision or mastectomy, the fresh tissue (without formalin treatment) was immediately transported to the Aberdeen Biomedical Imaging Centre (transportation time less than 5 min, Fig. 1). The ex vivo specimen was scanned in air and positioned in the isocentre of the scanner. The data were acquired on a 3 T whole-body clinical MRI scanner (Achieva TX, software version R5.1.7, Philips Healthcare, Best, Netherlands) using a body coil for uniform transmission and a 32-channel receiver head coil for high sensitivity detection. The DQF sequence was implemented locally using Philips Pulse Programming Environment (PPE). The T_1_-weighted anatomical images were acquired using standard 3D sequence with an isotropic voxel size of 1 mm, matrix size of 220 × 220, repetition time (TR) of 5.2 ms, echo time (TE) of 2.7 ms, imaging volume encompassing the specimen. Subsequently, the lactate spectrum was acquired from a single voxel snug-fit to the tumour using single voxel DQF PRESS sequence,^13^ with TR/TE of 1.25 s/144 ms, spectral editing frequency at 4.1 ppm and 512 averages. A reference spectrum was acquired from the same voxel using single voxel PRESS sequence with TR/TE of 1.25 s/144 ms, 16 averages. The total acquisition time for lactate spectrum was 11 min.

All spectra were processed following standard procedures and quantified using AMARES algorithm^18^ in the jMRUI software (v3.0, TRANSACT, Leuven, Belgium).^19^ Water and lactate amplitudes were quantified from reference and lactate spectra, respectively, using corresponding prior knowledge databases.^20^ The lactate concentration in the tumour was then computed from water and lactate amplitudes, literature breast tissue biochemical composition and relaxation properties on the 3 T scanner.^21^ The quantification of lactate concentration was conducted following written procedure and blinded from group allocation. Full details of quantification and spectra from breast tumour specimens are provided in Supplementary Information.

### Histopathological analysis

Upon the completion of scanning, the specimen was immediately transported to the Pathology Department at Aberdeen Royal Infirmary (transportation time less than five minutes, Fig. 1) for formalin fixation. Routine clinical histopathological examination was undertaken on the specimen to define the tumour characteristics (including size and grade) along with the nodal status for Nottingham Prognostic Index (NPI).^22^ Subsequently, immunostaining was performed in a single batch for Ki-67,^23^ LDH-A^24^ and LDH-B^25^ including appropriate positive controls. The immunostains were assessed semi-quantitatively by two pathologists independently (EH and IM), blinded from group allocation.

### Statistical analysis

All statistical analysis was performed in the SPSS software (Release 23.0, SPSS Inc., Chicago, IL, USA). Normality was determined on all the collected data using the Shapiro-Wilk test. Two-sample *t*-tests were performed on normally distributed data to assess difference between tumours with grade II and grade III histology, and Mann-Whitney *U* tests for non-normally distributed data. Fisher’s exact tests were applied to study the association between grades and other tumour pathological features. Pearson’s correlation test was performed between lactate concentration and tumour size, with Spearman’s rank correlation tests for lactate concentration against NPI, Ki-67 and LDH-A. A *p*-value < 0.05 was considered statistically significant.

## Results

The patient characteristics are shown in Table 1. All patient demographics, apart from NPI, were normally distributed. There were no significant differences in age, body mass index and tumour size between groups. Lactate was measurable in all spectra, defined as Cramér-Rao lower bound (CRLB) of the fit below 30%.

**Table 1:** Patient demography.

Characteristic	All (*n* = 30)	Grade II (*n* = 15)	Grade III (*n* = 15)	*p*-value
Age	61.1 ± 11.5	61.0 ± 12.0	61.2 ± 11.3	0.9628
Body mass index (BMI)	30.4 ± 6.4	30.4 ± 6.6	30.3 ± 6.6	0.9771
Tumour size (cm)	2.5 ± 0.8	2.4 ± 0.9	2.5 ± 0.7	0.7650
Tumour volume (cm^3^)^a^	20.0 ± 21.3	20.3 ± 19.2	19.6 ± 23.9	0.9335
Nottingham prognostic index (NPI)	4.41 (3.62–4.56)	3.62 (3.46–4.29)	4.50 (4.44–5.02)	0.0001*
*Tumour type*				
pTNM Stage				
I	5	3	2	1.000
II	25	12	13	
Lymphovascular invasion (LVI)	17	9	8	1.000
Lymph node involvement	8	4	4	1.000
Oestrogen receptor (ER+)	22	13	9	0.215
Human epidermal growth factor receptor 2 (HER2+)	6	2	4	0.651
Triple-negative breast cancer (TNBC)	7	1	6	0.080

Patient demography and clinical histopathological findings of excised breast tumours are shown for each group and the entire cohort. Quantitative data are expressed as mean and standard deviation (apart from Nottingham Prognostic Index where median and interquartile range are shown), while qualitative data expressed as number of positive cases.

*Significant findings (*p*  <  0.05) are marked.

^a^Tumour volume extracted from T_2_-weighted MRI.

**Fig. 2:**
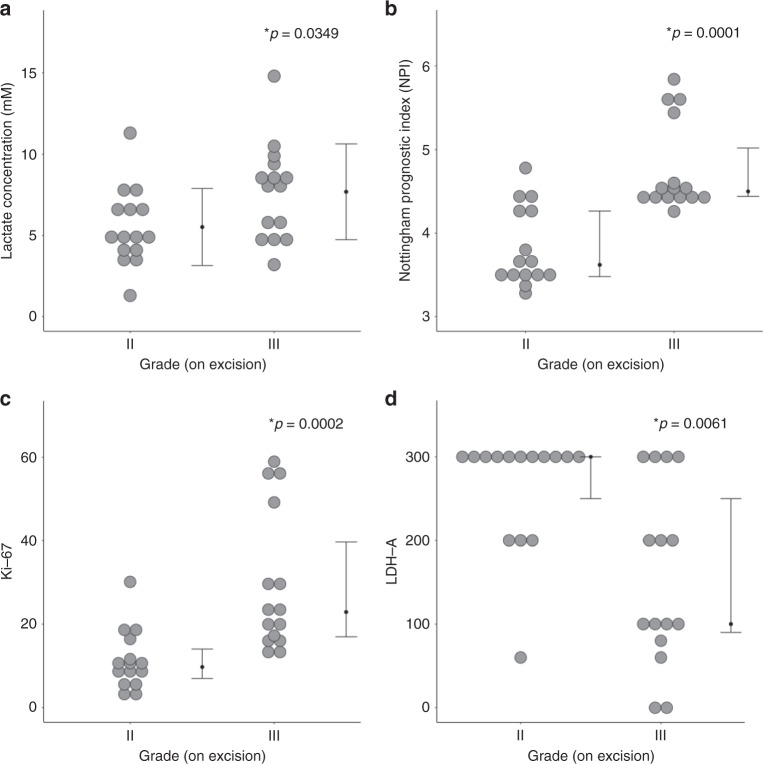
Lactate concentration, NPI and histological markers Ki-67 and LDH-A between grade II and III breast tumours. **a** Lactate concentration (*n* = 15), **b** Nottingham Prognostic Index (NPI) (*n* = 15), **c** Ki-67 (*n* = 15) and **d** lactate dehydrogenase A (LDH-A) expression (*n* = 15). Each dot represents the measurement obtained in each patient, and the dots are organised in two columns corresponding to the tumour grades. For lactate concentration, the error bar indicates the mean and standard deviation. For NPI, Ki-67 and LDH-A expression, it indicates the median and interquartile range. The two-tailed Student’s *t*-test was performed between the groups for lactate concentration, while Mann-Whitney *U* tests for NPI, Ki-67 and LDH-A expression. *P* < 0.05 was considered statistically significant and is marked by ‘asterisk’.

There was a significantly higher (*t* = 2.2224, *p* = 0.0349) lactate concentration in grade III (7.7 ± 2.9 mM) than in grade II (5.5 ± 2.4 mM) (Table 2, Fig. 2a). NPI was significantly higher (*z* = 3.819, *p* = 0.0001) in grade III (median: 4.50; interquartile range (IQR): 4.44–5.02) than in grade II (median: 3.46; IQR: 3.46–4.29) (Table 1, Fig. 2b). There was a significantly higher (*z* = 3.712, *p* = 0.0002) Ki-67 expression in grade III (median: 22.9%, IQR: 16.7–40.2%) than in grade II (median: 9.7 %, IQR: 6.3–16.4%) (Table 2, Fig. 2c). There was a significantly higher (*z* = 2.743, *p* = 0.0061) LDH-A expression in grade II (median: 300, IQR: 250–300) than in grade III (median: 100, IQR: 80–250) (Table 2, Fig. 2d). There was a significantly higher (*z* = 2.821, *p* = 0.0048) LDH-B expression in grade III (median: 40, IQR: 0–120) than in grade II (median: 0, IQF: 0–10) (Table 2).

**Table 2:** Lactate concentration, histological markers and prognostic indicator NPI.

	All (*n* = 30)	Grade II (*n* = 15)	Grade III (*n* = 15)	*t/z-*score, *p*-value	Correlation			
					LDH-A	LDH-B	Ki-67	NPI
					*ρ*-score, *p*-value	*ρ*-score, *p*-value	*ρ*-score, *p*-value	*ρ*-score, *p*-value
Lactate Concentration ([Lac]) (mM)	6.6 ± 2.9	5.5 ± 2.4	7.7 ± 2.9	*t* = 2.2224, *p* = 0.0349*	−0.3734, 0.0421*	0.3101, 0.0954	0.3041, 0.1023	0.3618, 0.0495*
*Immuno-histochemistry*								
LDH-A (0-300)	250 (100–300)	300 (250–300)	100 (80–250)	*z* = 2.743, *p* = 0.0061*	–	−0.7710, <0.0001*	−0.7258, <0.0001*	−0.4432, 0.0142*
LDH-B (0-300)	5 (0–50)	0 (0–10)	40 (0–120)	*z* = 2.821, *p* = 0.0048*	–	–	0.7893, <0.0001*	0.2419, 0.1978
Ki-67 (%)	16.6 (9.7–24.0)	9.7 (6.3–16.4)	22.9 (16.7–40.2)	*z* = 3.712, *p* = 0.0002*	–	–	–	0.4079, 0.0253*

Lactate concentration, lactate dehydrogenase A and B (LDH-A and LDH-B) expression and proliferative marker Ki-67 expression are shown for groups and the entire cohort. The spread of LDH-A, LDH-B and Ki-67 expression is reported as median and interquartile range (IQR). Correlation (Spearman’s *rho* (*ρ*)) scores of lactate concentration against histological markers and Nottingham Prognostic Index (NPI) are also shown.

*Significant findings (*p*  <  0.05) are marked.

**Fig. 3:**
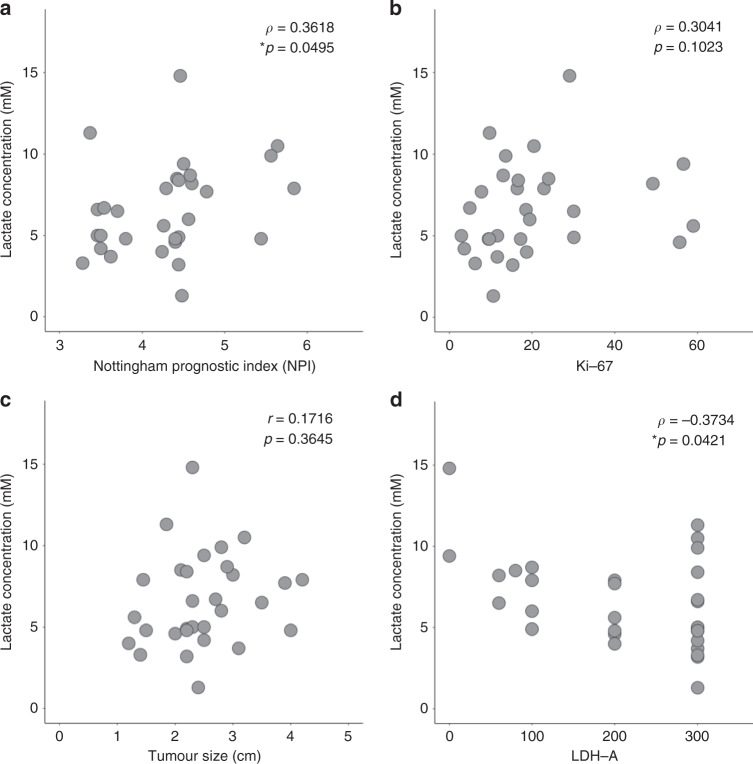
Association between lactate concentration and NPI, Ki-67, tumour size and LDH-A. Lactate concentration was correlated against **a** Nottingham Prognostic Index (NPI) (*n* = 30), **b** Ki-67 expression (*n* = 30), **c** tumour size (*n* = 30) and **d** lactate dehydrogenase A (LDH-A) expression (*n* = 30) within the entire cohort and shown as scatter plots. Spearman’s correlation tests (*ρ* score: **a**, **b**, **d**), Pearson’s correlation test (*r* score: **c**) were used and corresponding *p*-values are displayed. Statistically significant *p*-values (<0.05) are marked by ‘asterisk’.

There was a significant correlation between lactate concentration and NPI (*ρ* = 0.3618, *p* = 0.0495, Table 2, Fig. 3a). There were no significant correlations between lactate concentration against Ki-67 expression (*ρ* = 0.3041, *p* = 0.1023, Table 2, Fig. 3b) or tumour size (*r* = 0.1716, *p* = 0.3645, Fig. 3c). There was a significant negative correlation between lactate concentration and LDH-A (*ρ* = −0.3734, *p* = 0.0421, Table 2, Fig. 3d).

## Discussion

In this work, we found lactate concentration was significantly higher in grade III breast tumours compared to grade II. Increased lactate concentration was associated with worse NPI, not associated with tumour proliferative activity or tumour size, and negatively associated with LDH-A expression. Hence, lactate concentration derived from DQF MRS is a marker sensitive to tumour grades, with an association to prognosis.

Lactate concentration, observed in this study, is in agreement with findings in breast cancer cell culture,^26^ xenografted animal tumour models,^16^ ex vivo human brain tumours^27^ and brain tumours in patients.^12^ Since accumulated lactate alters the cellular physical appearance through the inflammatory activities and deregulation of immune function,^8^ the sensitivity to tumour grade is through the sub-scores associated with cellular appearance of tubule formation, nuclear pleomorphism and mitoses. Lactate concentration quantified over the entire tumour addresses the drawbacks in biopsy methods of intratumoural heterogeneity and partial sampling error. Lactate concentration is a quantitative objective measure compared to immunostaining methods and FDG-PET, reducing the demand on manpower and ambiguity. Hence, lactate concentration from DQF MRS is a non-invasive marker sensitive to tumour grade, with significant advantages over current methods for quantifying AG.

The correlation between lactate concentration and NPI indicates the prognostic value of lactate concentration in breast cancer, in line with elevated release of lactate in highly malignant breast cancer cells.^26^ Lactate supports advancing epithelial cancer cells as energy source,^6^ while macrophage function is severely compromised at high lactate concentration (>15 mM) with diminished production of natural killer cells and apoptosis of T cells.^8^ Lactate concentration does not show significant correlation with crude tumour size or Ki-67, underscoring lactate concentration as a specific marker to AG. Proliferative activity marker Ki-67 reveals the rate of division of cellular nuclear materials^28^ (that is possibly discordant with the mitotic index^29^), while lactate enhances the survival advantage of tumour cells reflecting invasion capability.^6^ The negative correlation against LDH-A was likely the result of feedback inhibition^30^ and depletion of pyruvate and nicotinamide adenine dinucleotide hydrogen (NADH)^31^ in higher grade tumour. The observed higher expression of LDH-A has been previously found in less aggressive 67NR breast cancer cells under hypoxia in preclinical model^16^ and more differentiated tumours in patients with gastric cancer,^32^ while observed higher LDH-B in higher grade is in agreement with previous studies in breast cancer.^33^ However, carbonic anhydrase IX (CA9), a strong prognostic marker, has been recently shown to induce an acidic tumour extracellular pH in vivo,^34^ decoupling lactate production regulated by LDH and lactate concentration. Hence, lactate concentration is a sensitive prognostic marker, specific to the invasion capability resulted from an acidic extracellular environment.

The lactate concentration obtained from single voxel approach is an average over the whole tumour, and does not inform the spatial distribution of lactate within tumour, limiting the accuracy in the cases with large disparity between tumour core and rim.^26^ Although triple-negative breast cancer is postulated to be more aggressive, there is no significant difference in lactate concentration between triple-negative (6 cases, mean: 8.4 mM) and non-triple-negative (nine cases, mean: 7.2 mM) in our grade III cohort, indicating no proven relation to this potential confounding factor. The deterioration of tissue after excision may affect the accuracy of lactate concentration quantification; however, the median time gap from excision to the middle of the DQF MRS scan was 25 min, significantly below the time frame for observing change in lactate concentration in postmortem tissues (3 h).^35^ The freshly excised tumour allows the use of a high sensitivity receiver head coil for improved measurement accuracy, while eliminating biological noise. Restriction of tumours to grade II and III with a size larger than 1 cm (24 specimens>2 cm) is to represent patient population likely receiving neoadjuvant therapy. The restriction of phenotype to invasive ductal carcinoma, the most common breast cancer, eliminates phenotype as a confounding factor, allowing adequate statistical power in a small patient cohort. The sample size is small; however, careful power calculation was conducted to assess the appropriate sample size^16^ before the evaluation of the lactate concentration from ex vivo tumours.

The current acquisition of lactate spectrum was long (11 min) and only suitable for lactate concentrations higher than 1.5 mM, demanding future development work to improve the sensitivity. Lactate was measurable in all spectra, and quantification was limited to lactate spectra with CRLB below 30%. The overlap of the distribution in lactate concentration between groups limits the prognostic value of lactate concentration obtained using current methodology in breast cancer. MRS is well known to have high specificity but low sensitivity. Future technological improvement including digital hardware and adaptive signal processing for phased array coils^36^ should be incorporated in further development to improve prognostic value. Future longitudinal studies on patients undergoing neoadjuvant chemotherapy will confirm the prognostic and treatment monitoring value of lactate concentration, and multicentre study with larger cohort size covering a complete range of phenotypes is essential for clinical adoption.

Lactate concentration from DQF MRS is a non-invasive marker sensitive to breast tumour grade and is associated with NPI as demonstrated in a cross sectional study. Lactate concentration provides a valuable clinical research tool for potential identification of treatment targets and application to improve image guided neoadjuvant chemotherapy.

## Supplementary information

Supplementary Information

## Data Availability

Data supporting this publication are stored at Institute of Medical Sciences and available upon request.
