# Supplementary material for: Lactate concentration in breast cancer using advanced magnetic resonance spectroscopy
Source: Br J Cancer. 2020 May 19;123(2):261–7. doi: 10.1038/s41416-020-0886-7 (PMC7374160; doi:10.1038/s41416-020-0886-7)
Supplement: Supplementary file 1 — Supplementary Information [file 41416_2020_886_MOESM1_ESM.pdf]

Supplementary Information

Lactate Quantification: All spectra were processed following standard procedures and quantified using AMARES algorithm [1] in the jMRUI software (v3.0, TRANSACT, Leuven, Belgium) [2]. Water and lactate amplitudes were quantified from reference and lactate spectra respectively using corresponding prior knowledge databases [3]. The lactate concentration was computed from lactate and water amplitudes using the following formula [4]:

$$[Lac] = \frac{n_{water}}{n_{Lac}} \frac{S_{Lac}}{S_r} [r]\, C_{T1,T2} \qquad \text{(F-1)}$$

where n_{water} and n_{Lac} denote the numbers of ^{1}H nuclei for each molecule, S_r the water amplitude, S_{Lac} the lactate amplitude, [r] the water concentration based on 82% water content [4], and $C_{T1,T2}$ the correction factor for T_1 and T_2 differences between lactate and water as:

$$C_{T1,T2} = \frac{[1 - \exp(-\mathrm{TR}/T1_{water})]}{[1 - \exp(-\mathrm{TR}/T1_{Lac})]} * \exp\left[TE\left(\frac{1}{T2_{Lac}} - \frac{1}{T2_{water}}\right)\right] \qquad \text{(F-2)}$$

The T_1 and T_2 values used for lactate quantification are detailed as follows. Water T_1 and T_2 in tumour were estimated to be 1200 ms and 65 ms respectively as the mean of published literature values [5,6]. Lactate T_1 and T_2 in tumour were estimated to be 1600 ms and 160 ms respectively as the mean of published literature values in MCF-7 and BT-474 breast tumours [7]. For small temperature variations and in the range between room and physiological temperature, T_1 and T_2 increase linearly with absolute

temperature (0K = -273°C, on the order of 1%/°C) [8-10]. All the specimens were scanned at room temperature (maintained at 22°C), eliminating the variation in quantification arising from temperature induced T_1 / T_2 fluctuation. The literature T_1 / T_2 values were obtained from human subjects / mice at 37°C and no adjustments were made for lactate concentration at room temperature. The quantification of lactate concentration was conducted following written procedure, and blinded from group allocation. The quality of lipid suppression and truthful detection of lactate is shown in Figure S1, and lactate spectrum from a patient with invasive breast carcinoma is shown in Figure S2 as a natural consequential step of this work. We performed four *in vivo* scans, and the major drawbacks were the limited sensitivity of the receiver breast coil (16-channel) and partial volume effect subsequent to patient movement. The acquisition time for *in vivo* scans was the same as in *ex vivo*, and provided sufficient signal-to-noise ratio for accurate lactate quantification.

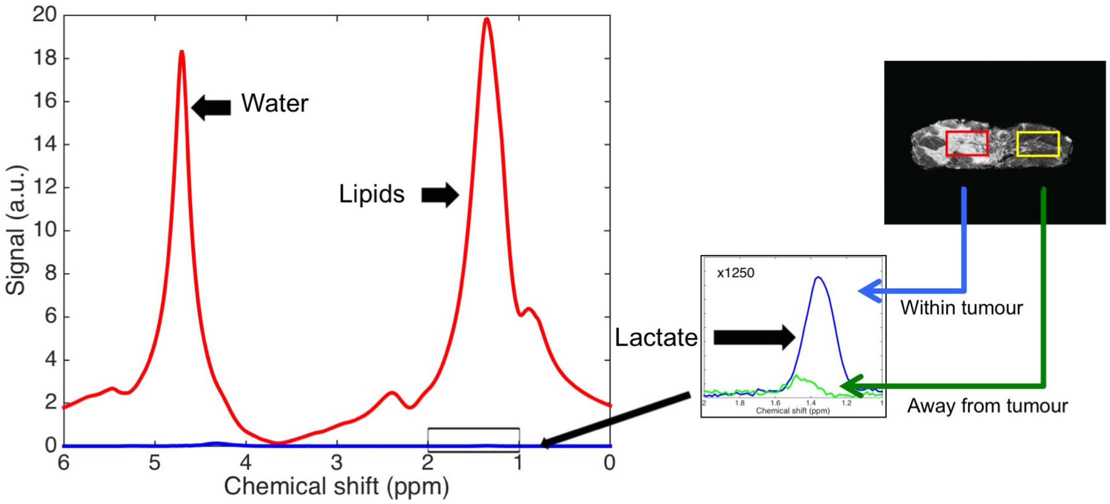

Figure S1. Lactate detection efficiency and lipid suppression with and without DQF spectroscopy in a mastectomy specimen.

Lactate DQF (blue) and reference spectrum (red) obtained from the tumour (red voxel) were co-registered on the same scale. In the reference spectrum, lactate cannot be detected due to dominant co-resonance lipid signal. A magnified [1, 2] ppm region of the lactate DQF spectrum from tumour shows clear lactate detection (blue), whereas a separate lactate DQF spectrum from adipose tissue (yellow voxel) shows no lactate (green).

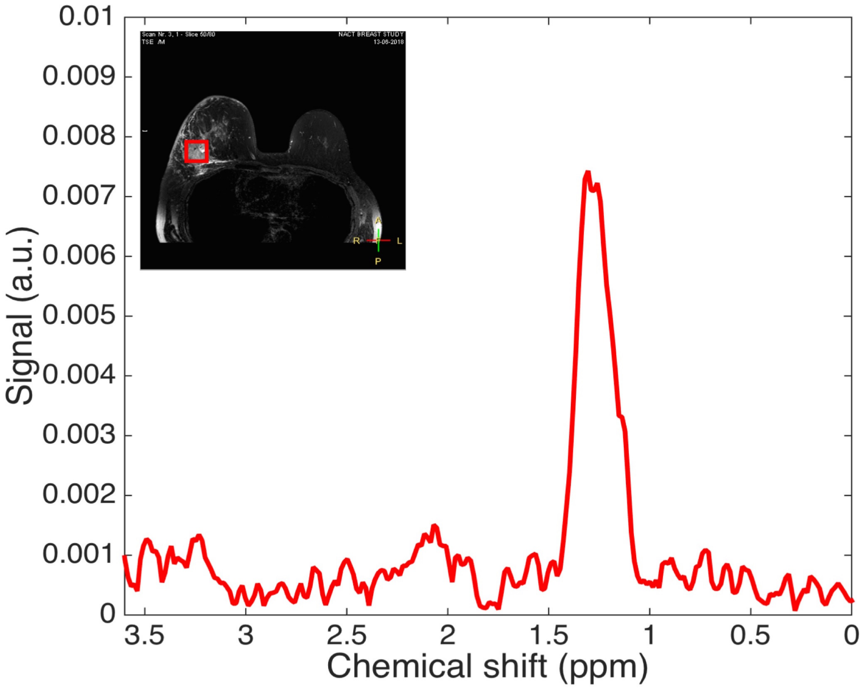

Figure S2. Lactate spectrum acquired *in vivo* from a patient with Grade III invasive breast carcinoma.

Lactate spectra acquired in a pilot study in patients with invasive breast carcinoma as a consequential step of this work following the demonstration of clinical utility of lactate concentration as a marker relevant to aerobic glycolysis. There was a prominent lactate peak (doublet at 1.3 ppm) inside the breast tumour (red voxel) in the patient.

References:

1. Vanhamme, L. & van den Boogaart, A. Improved method for accurate and efficient quantification of MRS data with use of prior knowledge. *J Magn Reson* **129,** 35–43 (1997).
2. Naressi, A., Couturier, C., Castang, I., de Beer, R. & Graveron-Demilly, D. Java-based graphical user interface for MRUI, a software package for quantitation of in vivo/medical magnetic resonance spectroscopy signals. *Comput Biol Med* **31,** 269–286 (2001).
3. Payne, G. S., Harris, L. M., Cairns, G. S., Messiou, C., deSouze, N. M., Macdonald, A., et al. Validating a robust double-quantum-filtered ^{1}H MRS lactate measurement method in high-grade brain tumours. *NMR Biomed* **29,** 1420–1426 (2016).
4. Sijens, P. E., Dorrius, M. D., Kappert, P., Baron, P., Pijnappel, R. M., Oudkerk, M. Quantitative multivoxel proton chemical shift imaging of the breast. *Magn Reson Imag* **28,** 314–319 (2010).
5. Bolan, P. J., Meisamy, S., Baker, E. H., Lin, J., Emory, T., Nelson, M., et al. In vivo quantification of choline compounds in the breast with ^{1}H MR spectroscopy. *Magn Reson Med* **50,** 1134–1143 (2003).
6. Edden, R. A. E., Smith, S. A. & Barker, P. B. Longitudinal and multi-echo transverse relaxation times of normal breast tissue at 3 Tesla. *J Magn Reson Imag* **32,** 982–987 (2010).
7. Annarao, S., Thomas, K., Pillarsetty, N., Koutcher, J. A. & Thakur, S. B. In vivo lactate T_1 and T_2 relaxation measurements in ER-positive breast tumours using SS-SelMQC editing sequence. In: Proceedings of the 19th Annual Meeting of ISMRM, Montréal, Canada. **19,** 3158 (2011).

8. Baron, P., Deckers, R., Knuttel, F.M., Bartels, L.W. T_1 and T_2 temperature dependence of female human breast adipose tissue at 1.5T: groundwork for monitoring thermal therapies in the breast. *NMR Biomed* **28**, 1463 – 1470 (2015).

9. Bottomley, P.A., Foster, T.H., Argersinger, R.E., Pfeifer, L.M. A review of normal tissue hydrogen NMR relaxation times and relaxation mechanisms from 1-100 MHz: dependence on tissue type, NMR frequency, temperature, species, excision, and age. *Med Phys* **11**, 425 – 448 (1984).

10. Rieke, V. & Butts Pauly, K. MR thermometry. *J Magn Reson Imag* **27**, 376 – 390 (2008).
